# Editorial: Cognitive-Motor Interference in Multi-Tasking Research

**DOI:** 10.3389/fpsyg.2019.01744

**Published:** 2019-07-30

**Authors:** Karen Zentgraf, Hermann Müller, Eliot Hazeltine

**Affiliations:** ^1^Department of Movement Science and Training in Sports, Goethe University Frankfurt, Frankfurt, Germany; ^2^Institute of Sports Science, Justus Liebig University Giessen, Nemolab, Germany; ^3^Department of Psychological and Brain Sciences, The University of Iowa, Iowa City, IA, United States

**Keywords:** interference, multi-tasking, costs, movements, real world

Multitasking is ubiquitous in our everyday life. Accordingly, situations in which two or more tasks need to be handled concurrently or in close temporal succession have been studied intensely. Different paradigms have been developed in that context (Koch et al., 2018). Over the last decades, the psychological refractory period (PRP) paradigm has dominated dual-task research, because it allows quantitative predictions of reaction time increases coupled to stimulus onset asynchrony. Part of the success of this paradigm is grounded in the fact that most of the studies are run under strict experimental control with very elementary tasks, mostly characterized by a definite start and ending. However, it remains unclear whether these limited settings sufficiently reflect the range of eventualities we find in real life. Rather, there is accumulating evidence that important factors modulating multitask performance are not sufficiently captured by the PRP approach. Here we focus on evidence that motor responses that involve continuous interaction with the environment may engage processes that alter the coordination of concurrently performed tasks in fundamental ways.

The studies collected in this Research Topic contribute to this question by showing that:

- Even basic postural tasks require central processing capacities, potentially competing against concurrent cognitive tasks.
- Movements in space are related to concepts of location and direction, thereby emphasizing aspects of spatial compatibility and embodied contingencies.
- Multitasking performance is not driven strictly by the set of stimuli and responses but rather depends on task representation within the subject.
- In cases in which postural control is required, task prioritization becomes a crucial factor. Irrespective of instruction, priority is given to tasks with larger costs of failure. Several studies presented here confirm that this effect is more pronounced in elderly persons.
- Although priority is often given to motor tasks, they still demonstrate dual-task interference. Surprisingly, there are also cases of a “dual-task benefit.”.
- The concept of “automaticity” must be (re)considered as a potential explanation for variations in dual-task costs.
- Motor behavior is generally not temporally discrete but evolves over time. The ability to predict changes in processing demands allows the control system to appropriately allocate resources.
- Dual-task settings push the control system to its limits, which makes them particularly useful to study control in clinical populations.

The study by Stelzel et al. nicely demonstrates aspect (A). Individual differences in dual-task performance in a motor-cognitive task can be explained by different degrees of involvement of the lateral prefrontal cortex, a region thought to play an important role in central resource allocation.

Aspect (B) is addressed by Stephan et al. Switch costs, mixing costs, and congruency effects are typically preserved under different postural demands. However, the authors observed an increased congruency effect when standing compared to sitting.

The study of Halvorson and Hazeltine links aspects (B) and (C). Previous studies have shown that dual-task costs are largely reduced when stimulus and response modalities are compatible within each task and separate across tasks. The authors show that this is not sufficient for the reduction of dual-task costs but that dual-task costs depend on the relationship between the tasks.

Also relating to aspect (C), Hosang et al. did not observe any modulation of the PRP-effect by hand-proximity to stimuli. The authors interpret this observation as confirmation that the bottleneck is in a central processing stage, which is not affected by peripheral (embodied) contingencies.

In line with (C), Schumacher et al. demonstrate that dual-task effects are not strictly linked to the sheer number of stimuli and responses on a given trial but critically depend on whether the task is represented as single task or dual task.

Aspect (D) emphasizes that task prioritization depends on the nature of the motor output. According to the “posture first” hypothesis (Lindenberger et al., 2000), postural tasks like balancing, walking, or running receive priority for processing resources due to the large costs of failure. Because costs of failure are higher in older subjects, their resource allocation is even more biased.

This is nicely supported by the study of Wechsler et al. Older subjects keep larger safety margins in a virtual driving scenario than younger participants. This effect is amplified under dual-task conditions, particularly when the secondary task requires visual attention.

However, Janouch et al. demonstrate that this “costly-task-first” effect is not pervasive. As expected, in an ecologically more valid street crossing scenario, dual-task costs increased with age. However, task prioritization did not follow a general “posture first” principle. Furthermore, dual-task costs were not consistently larger for visual than for auditory versions of the loading task. The priority given to each task appears to be specific to the circumstances.

This specificity is confirmed in a study with children by Schott and Klotzbier. They observe an interaction between task demands with age and discuss these findings in light of a resource model that assumes that allocation regimes and executive function resources differ across age groups.

Kiss et al. did not find any signs of dual-task costs when combining a cognitive task (counting) and a motor task (balancing), providing a link between aspects (C) and (E). Furthermore, single-task practice only improves the practiced task, whereas dual-task training improves both tasks. This finding is taken as an indicator for no or very small overlap in processing for the tasks.

Lüder et al. also use a cognitive-motor paradigm and demonstrate that task prioritization may change with age (D). In their study, children show performance decrements in standing and walking when a calculation task is added (E). These costs are reduced similarly by single and dual-task training.

But even the movements of experts (i.e., athletes) demonstrate that posture is not always preserved from performance decrement in case of cognitive-motor task interference. Fleddermann and Zentgraf show that jumping performance of elite volleyball players show clear decrements when jumping was linked to a game-specific, visually presented decision task (E).

However, performing a motor and a cognitive task in parallel does not necessary lead to impairments (dual-task costs) in all relevant performance measures (F). Langhanns and Müller demonstrate that the frequency of repetitive movements sometimes increases when a cognitive task is performed concurrently. However, this seems to be limited to motor tasks that are under automatic control.

In these cases, processing load may be considerably reduced, partly because events are predictable (G). Accordingly, Broeker et al. show that processing in multitasking is altered depending on the degree of predictability of events. Prediction of the time course of events allows for the preplanned allocation of processing resources, to prepare for upcoming trials but also for error processing and updating the contents of memory. It is argued that these predictive processes are automatic but also depend on task characteristics and explicit cues.

Ewolds et al. combined a go/no-go auditory RT task with a motor tracking task to reveal differences in processing load when the tracking task was partly predictable. Differences in predictability might be induced by either implicit or explicit knowledge about regularities in the target trajectory. Even though the effects of implicit/explicit predictability are visible in motor performance, dual-task costs are small and therefore not a major target of this manipulation.

Besides these contributions to deepening our understanding of cognitive control, studying multitasking might also contribute to addressing diagnostic problems in clinical contexts (G). McIsaac et al. point to the fact that dual-task costs are indicative of limitations in processing capacities in healthy individuals and thus may be exacerbated in neurodegenerative patients. This group of persons may benefit strongly if specific dual-task-impairments could be addressed by specific interventions.

This Research Topic develops new areas in multitasking research and attempts to evolve the field with respect to traditional concepts. We have proposed theoretical and empirical challenges that these new and traditional paradigms present to multitasking research. In this editorial, we provide a brief inventory of the papers in the Research Topic and outline promising avenues for future research. Specifically, we highlight the role of the motor components of responses and how these components are embedded within a task context in determining the pattern of dual-task costs. Understanding these factors is essential for generating models of dual-task performance that translate to real-world situations.

## Author Contributions

All authors listed have made a substantial, direct and intellectual contribution to the work, and approved it for publication.

### Conflict of Interest Statement

The authors declare that the research was conducted in the absence of any commercial or financial relationships that could be construed as a potential conflict of interest.
